# The Online Health Information Needs of Family Physicians: Systematic Review of Qualitative and Quantitative Studies

**DOI:** 10.2196/18816

**Published:** 2020-12-30

**Authors:** Piet van der Keylen, Johanna Tomandl, Katharina Wollmann, Ralph Möhler, Mario Sofroniou, Andy Maun, Sebastian Voigt-Radloff, Luca Frank

**Affiliations:** 1 Friedrich-Alexander University Erlangen-Nürnberg Institute of General Practice University Hospital Erlangen Erlangen Germany; 2 Institute for Evidence in Medicine, Medical Center Faculty of Medicine University of Freiburg Freiburg Germany; 3 Cochrane Germany Foundation Freiburg Germany; 4 Institute for Health Services Research and Health Economics Heinrich-Heine-University Düsseldorf Center for Health and Society, Faculty of Medicine Düsseldorf Germany; 5 Division of General Practice Medical Center, Faculty of Medicine University of Freiburg Freiburg Germany

**Keywords:** family physicians, general practitioners, primary care, needs, barriers, online information, health information, health resources, internet, information-seeking behaviors, mobile phone

## Abstract

**Background:**

Digitalization and the increasing availability of online information have changed the way in which information is searched for and retrieved by the public and by health professionals. The technical developments in the last two decades have transformed the methods of information retrieval. Although systematic evidence exists on the general information needs of specialists, and in particular, family physicians (FPs), there have been no recent systematic reviews to specifically address the needs of FPs and any barriers that may exist to accessing online health information.

**Objective:**

This review aims to provide an up-to-date perspective on the needs of FPs in searching, retrieving, and using online information.

**Methods:**

This systematic review of qualitative and quantitative studies searched a multitude of databases spanning the years 2000 to 2020 (search date January 2020). Studies that analyzed the online information needs of FPs, any barriers to the accessibility of information, and their information-seeking behaviors were included. Two researchers independently scrutinized titles and abstracts, analyzing full-text papers for their eligibility, the studies therein, and the data obtained from them.

**Results:**

The initial search yielded 4541 studies for initial title and abstract screening. Of the 144 studies that were found to be eligible for full-text screening, 41 were finally included. A total of 20 themes were developed and summarized into 5 main categories: *individual needs* of FPs before the search; *access needs*, including factors that would facilitate or hinder information retrieval; *quality needs* of the information to hand; *utilization needs* of the information available; and *implication needs* for everyday practice.

**Conclusions:**

This review suggests that searching, accessing, and using online information, as well as any pre-existing needs, barriers, or demands, should not be perceived as separate entities but rather be regarded as a sequential process. Apart from accessing information and evaluating its quality, FPs expressed concerns regarding the applicability of this information to their everyday practice and its subsequent relevance to patient care. Future online information resources should cater to the needs of the primary care setting and seek to address the way in which such resources may be adapted to these specific requirements.

## Introduction

### Background

Digital transformation and the ubiquitous availability of online information resources have diversified the process of obtaining and evaluating information in general. Although information availability has seen a transition from classical sources of information to digital equivalents, it has brought with it new barriers such as technical requirements, paying systems, or the need for paid membership to access certain contents. Following initial work on the information needs of doctors by Covell et al [1], reviews have summarized the needs, information-seeking behaviors, and resources used to answer clinical questions that have arisen from everyday practice [2-12]. One reason for clinicians to conduct an information search is to answer questions arising from their daily practice and patient care [12]. Doctors therefore frequently use the internet for professional purposes [13]. They encounter evermore internet-informed patients, who bring information into the consultation [14] and use the internet as their preferred source of health information [15,16]. This inevitably affects the doctor-patient interaction [17] and health-related decision making [18]. The variety and abundance of online medical information may be overwhelming when it comes to critically appraising and evaluating the quality of these resources [19]. Doctors thus face new challenges when it comes to the utilization and adoption emerging through digital transformation in health care. The following questions should be raised: What reasons, facilitators, and barriers exist for doctors during online information searches? How are their information needs and information-seeking behaviors may be affected by digital transformation? Despite the body of available literature, we identified 3 gaps in the literature on the information needs among doctors, leading us to conduct this systematic review:

1. Not family physician (FP)–specific: Half of the systematic reviews examined were not specific to primary care or FP, but on the information needs of doctors in general [4,7,9,10,12], whereas reviews addressing the information needs and information-seeking behaviors of FPs are outdated [2,3,5,6,8]. The latest review by Clarke et al [11] in 2013, analyzed the information-seeking behavior of FPs, trainees, nurse practitioners, nurses, and nurse coordinators in a combined review to better understand clinical decision making. As the daily routines of medical specialties differ greatly, so do the respective information needs of doctors [9], which arise from tackling specific clinical tasks in everyday practice [20]. FPs are confronted with diverse clinical questions and therefore may have differing information requirements than other specialist colleagues. These requirements could be met with a multitude of available online tools and software systems developed within the recent decade. To the best of our knowledge, no recent systematic review has exclusively examined the information needs of FPs toward online health information.
2. Out of date: One review from 2011 studied information needs and information-seeking behaviors of hospital-based doctors compared with primary care physicians regarding the access of electronic information [21]. However, technological advancements as well as rapidly changing information delivery systems over the last decade have altered information retrieval in general and specifically in the health care setting.
3. Contradictory evidence: It remains questionable whether the perceived needs of doctors reflect their actual needs [9]. Existing, but not perceived, needs of physicians could remain unexpressed. The analysis of barriers and facilitators before, during, and after the information search itself may give insights into existing but unperceived or unexpressed needs [9].

### Objectives

This systematic review asks “What needs, demands, barriers and facilitators exist for FPs to search for online health information?” We intend to fill these gaps in the literature and aim to do the following:

1. Review studies that analyze the information needs and information-seeking behaviors of FPs in the primary care setting.
2. Focus on online information retrieval by considering the technological advancements in health care and medical information over the last 20 years.
3. Include factors that facilitate or hinder the need for and retrieval of online information in the FP setting.

Therefore, this study intends to summarize the 3 elements of information need, literature searches, and resources as they are interlinked, as suggested by Davies [9].

## Methods

### Methodological Approach

We performed a systematic review.

### Search Strategy

We searched for relevant studies using MEDLINE (via PubMed), Web of Science Core Collection (SCI-Expanded), and Scopus. Furthermore, the reference lists of identified primary papers were screened to identify other potentially relevant citations. The initial search in all databases was performed on May 2, 2018, and updated on January 21, 2020. We formed a search strategy in cooperation with Cochrane Germany and a consulting medical colleague. We started an explorative search term comprising “physician* AND health information AND need.” For the specifications of the explorative search strategy, we searched for relevant synonyms and corresponding Medical Subject Headings (MeSH) terms to extend the explorative terms. These were matched with *MeSH On Demand* and with MeSH terms in similar papers, retrieved by using the explorative search. We established generated blocks for each aspect of the review question, such as the participants involved, the areas of interest, and the setting. Synonyms or similar MeSH terms within each block were combined with the OR operator. The blocks were then combined with the AND operator. We limited our search to the years spanning 2000 to 2020 for the following reasons: global internet access was only widely available from the year 2000 onwards. Subsequently, increased use of the internet could be assumed. This period covers milestone technical developments, such as broadband and mobile internet access, smartphone development and the accompanying hardware and software changes, and social media utilization. Before 2000, only about 5% [22] of the world population had internet access. Thus, it seems reasonable to limit the timeframe accordingly. The final search terms and search details used are provided in Multimedia Appendix 1.

### Inclusion and Exclusion Criteria

We included original qualitative, quantitative, and mixed methods studies, which assessed the needs of FPs and their requirements for online health information, regardless of the medical indication. These studies included those that assessed these needs explicitly or more implicitly measured requirements, barriers, and demands in asking clinical questions during an FPs’ working day or during continuous medical education (CME) programs. If studies addressed a variety of professions or specialties, we only considered those that consisted of at least 50% FPs in the study population. We included studies retrieved by the search in German and English only, regardless of the impact factor, peer-review process, or publishing process (eg, book, journal, and dissertation).

We excluded the following:

- Reviews, conference proceedings, evidence syntheses, editorials, commentaries, study register entries, protocols, or works that were unobtainable.
- Studies conducted in developing countries that had a very different or underdeveloped health care system, if a reasonable comparison with the primary care systems of the included studies (eg, Germany, the United Kingdom, and France) was not feasible.
- Double publications that only received minor edits or updates to the initial study, by choosing those with the most complete data set.

Furthermore, studies were deemed to be ineligible if:

- The wrong type of information was addressed: As this review aimed at the needs of FPs in obtaining online health information, we excluded:
  Studies that only analyzed health information in exclusively printable (nononline) formats.
  Studies that focused on electronic health records or systems aiming at patient information.
- The wrong population was addressed: patients, general public, or nonmedical health professionals (eg, studies that addressed evaluation or utilization of patient-centered telephone or online counseling, interactive apps, online forums, social media, or patient portals with protected log-ins or personalized patient data).
- Only outcomes not connected to information-seeking behavior or information needs toward online health information were measured (eg, piloting and evaluation of specific knowledge interventions or educational programs for physicians).

### Study Selection

All duplicates were removed automatically using Endnote X9.3.3 (Clarivate) and subsequently by hand. Study selection was performed with Covidence [23], which is specifically designed for Cochrane reviews and frequently used for review management [24,25]. Two researchers (LF and PK) independently screened titles and abstracts and excluded studies that were not eligible. LF and PK then independently screened full-text copies of potentially relevant papers, excluding any studies that were not eligible and documenting the reasons for exclusion. Any disagreements in any phase were resolved through discussion and consensus. Due to the broad themes of independently screened papers, we did not measure inter-rater reliability (kappa) because a quantitative measurement of agreement would not have reflected the qualitative consensus process.

### Data Extraction

**Figure 1:**
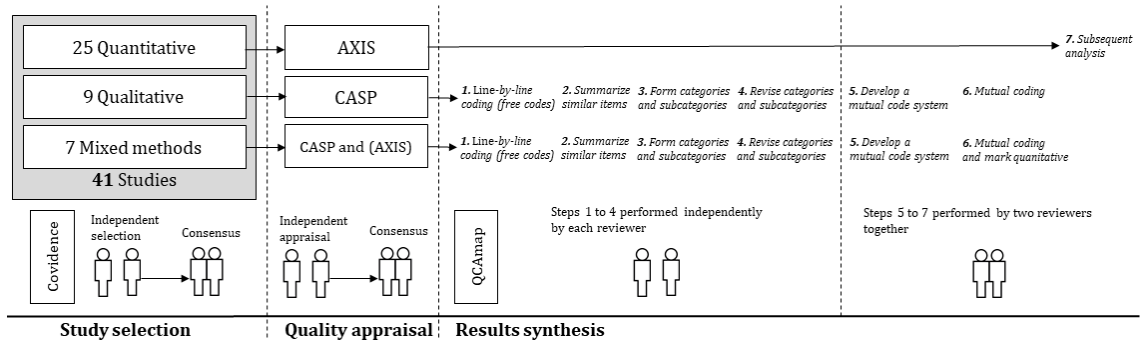
Methods overview for study selection, quality appraisal, and results synthesis (for details see text). AXIS: appraisal tool for cross-sectional studies; CASP: critical appraisal skills program.

Two reviewers developed and piloted a data extraction form independently and manually and obtained the following study characteristics: authors, publication date, title, study type (quantitative, qualitative, or mixed methods), type of data collection (questionnaires, interviews, etc), recruitment of participants (email, hospital, etc), number of FP participants, indication for the health information addressed, the type of health information (online, app, etc), and the outcome variables (needs and requirements). The reviewers resolved any disagreements in the extraction phase through discussion and consensus. An overview of the data extraction phase, including quality appraisal, results analysis, and synthesis, is displayed in Figure 1.

#### Quality Appraisal of the Studies

The quality of the studies was assessed by using 2 instruments. For qualitative studies, we applied the critical appraisal skills program (CASP) checklist [26]. For quantitative studies, we applied the appraisal tool for cross-sectional studies (AXIS) tool [27]. For mixed methods studies, we applied the CASP checklist and, if applicable, the AXIS tool. CASP offers distinct and easy-to-use checklists for critically appraising different forms of evidence and studies adapted from the *JAMA Users Guide to the Medical Literature* [28] and is used in similar reviews [29]. The AXIS tool is a new appraisal tool for cross-sectional studies, currently cited in more than 60 reviews as well as in a recent systematic review assessing the effectiveness of apps [30]. As many of the included quantitative studies had a cross-sectional design, it seemed suitable for this review. AXIS offered no numerical scale to assess quality, but instead aimed to assess the individual characteristics of a study and therefore seemed suitable, as quantitative data were too heterogeneous to perform meta-analyses. Critical appraisal also served as an indicator of possible strengths and weaknesses of the studies and any possible implications for thematic synthesis. All quality aspects were extracted independently by 2 reviewers (LF and PK) in a separate data sheet and were later combined by discussion and consensus into the tables shown in Multimedia Appendices 2 [31-46] and 3 [21,32,34,36,40,47-70].

#### Analysis and Synthesis of Results

A structured analysis of all included studies as well as thematic synthesis was performed based on the method described by Thomas and Harden [71] and according to a systematic review by Möhler and Meyer [72]. All full texts, figures, tables, and supplementary materials of the included studies were uploaded to a qualitative content analysis software (QCAmap), according to Mayring [73]. In studies where mixed populations were addressed, but FPs made up at least 50% of the study population, we only extracted qualitative and quantitative data if they were represented separately. They were excluded from the study if data regarding FPs were not separately extracted. Data synthesis was performed in 7 stages (Figure 1):

1. The results of the qualitative and mixed methods studies were coded line by line according to the meaning of the content (free codes). The free codes were named as per *the description of the item*, enabling the reader to identify the way in which an item was defined in the text of the included studies.
2. A superior item name was generated to summarize the descriptions of similar items.
3. Item names were organized into related areas of descriptive themes (main categories and subcategories) by inductive category formation [73].
4. These categories were compared within the studies to analyze similarities or differences, or create new categories, if existing ones were insufficient. Stages 1 to 4 were performed by both reviewers (LF and PK) independently, thereby generating 2 separate code systems.
5. Both reviewers compared and discussed their respective code systems to achieve consensus on a mutual code system.
6. With this mutual code system, the 2 reviewers together again coded all full-text papers line by line, resulting in a more general and objective coding system during text interpretation. In mixed methods studies, where quantitative content showed relevant connections to qualitative content, this content was additionally marked with a quantitative code to later allow linking to both qualitative and quantitative data sets.
7. Purely quantitative studies were subsequently analyzed to support the main categories and subcategories derived from qualitative synthesis in a sequential synthesis design [74] and without conducting meta-analyses.

During the collection of the main categories, this study observed that *needs* could be expressed directly and explicitly (eg, *need for reduced information*), or more indirectly (implicitly), by naming distinct barriers (eg, *the overabundance of information*), perceived lacks (eg, *lack of reduced information*), and possible facilitators (eg, *suggesting less information*) during the information search. Where those diverse expressions occurred, they were summarized under the same category to enable the compilation of different aspects of utterance meaning [75]. Furthermore, categories were summarized by acknowledging information search and seeking behavior as a process [9], beginning with a personal need, leading to the access of the information, utilizing it, and the implications of the information used. Inevitably, emerging categories can overlap in certain aspects.

## Results

### Flow Diagram

**Figure 2:**
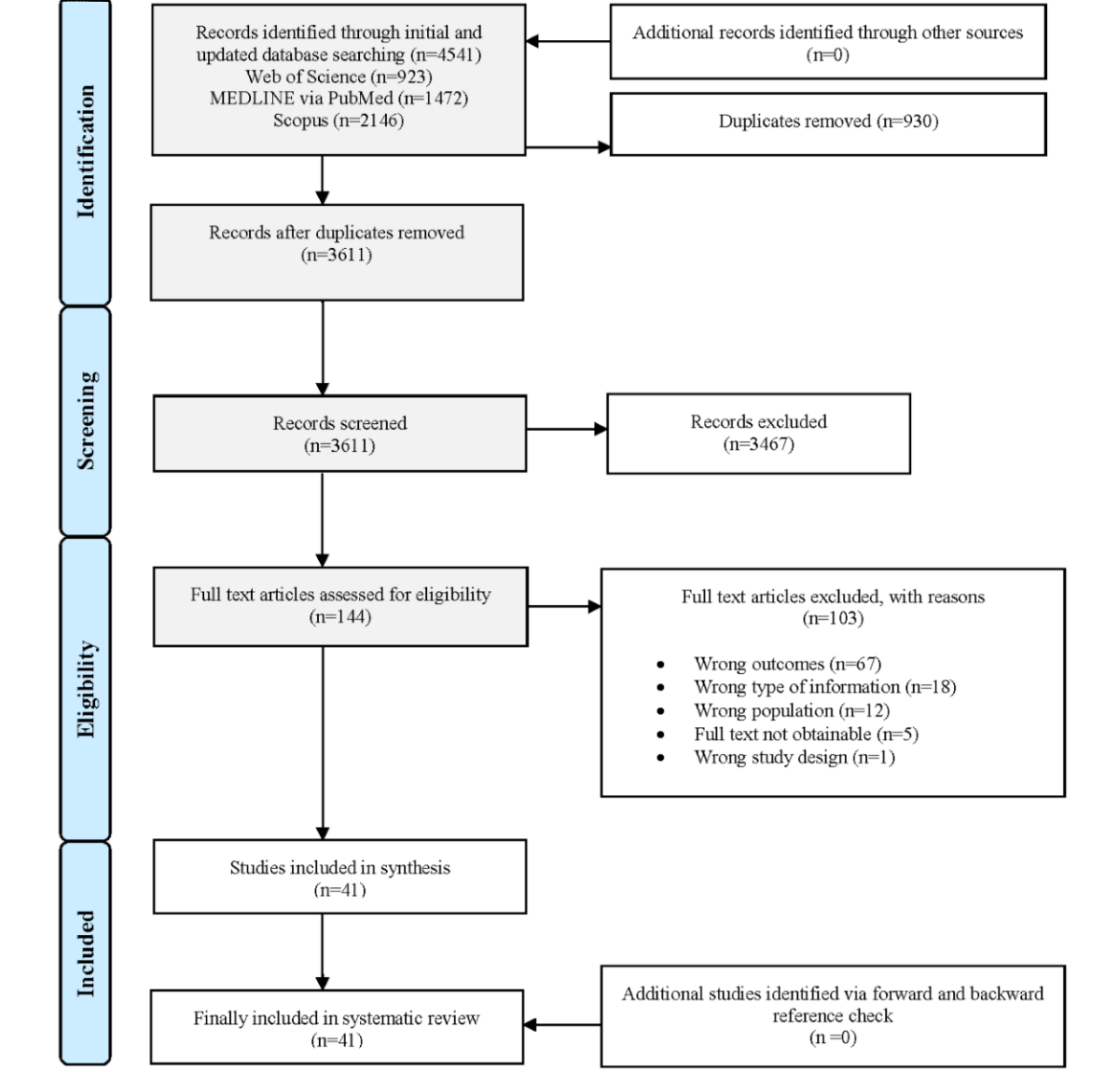
Flow diagram.

After deduplication, the search retrieved 3611 citations. A total of 144 publications were screened in full text, and 103 publications were excluded. We included 41 studies in the synthesis. The study flow is shown in the preferred reporting items for systematic reviews and meta-analyses [76] (PRISMA) diagram (Figure 2).

### Characteristics of Included Studies

#### Type of Studies and Data Collection

Out of the 41 included studies, 25 were quantitative studies, 9 were qualitative, and 7 were mixed methods studies. Data were collected through a survey (n=18); interview (n=5); questionnaire or logbook (review of medical notes and consultation records; n=5); collection of clinical questions by interview or observation (n=4); combined survey, interview, or focus groups (n=4); focus groups alone (n=3); or as a prospective study with electronic data collection (n=2). For a detailed overview of qualitative and mixed methods studies as well as the aims of quantitative studies, their characteristics, recruitment settings, and the outcomes formulated, see Tables 1 and 2.

**Table 1:** Characteristics of qualitative and mixed methods studies.

Author (Reference)	Aims	Data collection	Data analysis	Sample size	Main results and themes	Type of information
Badran et al 2015 [31]	Explore the viewpoint of FPs^a^ on advantages and disadvantages of educational email alerts	Interview	Thematic analysis	15 FPs	Advantages or disadvantages of educational email alerts; knowledge, attitude, and behavior toward email	Email or educational email alerts
Barrett et al 2004 [32]	No clear aim stated	Survey and interview	Descriptive design	88 GPs^b^ (survey); 15 GPs (interview)	PDA usage by residents; advantages or disadvantages of PDA use	Information regarding the use of PDAs
Boissin 2005 [33]	Consider the use of computers by GPs, analyze the impact of computerization on information-seeking behavior	Interview	N/A^c^	32 GPs	Computerization; opinions about new technologies; the GPs working environment; information behavior	Internet information
Bryant 2004 [34]	Explore factors that motivate GPs to pursue information	Survey interview and focus group	N/A	19 GPs (interview); 39 GPs (focus group)	Information needs and seeking; preferences; attitudes toward libraries; information sources used	Mixed
Cook et al 2013 [35]	Understand barriers and enabling factors influencing physician point-of-care learning and decisions physicians are facing	Focus group	Grounded theory	50 PCPs^d^	Barriers and enabling factors of point-of-care learning	Mixed
Cullen 2002 [36]	Determine the extent of use of the internet for clinical information among FPs	Survey and interview	N/A	294 GPs (survey); 20 GPs (interview)	Use of internet; information sources; type of information sought; search skills; use of critical evaluation of information and impact on decision	Internet information
Ely et al 2000 [37]	Develop a taxonomy of doctors’ questions about patient care	Collection of clinical questions (interview)	Observation and text analysis	103 FPs	Classify clinical questions	Clinical questions
Ely et al 2002 [38]	Describe obstacles encountered when attempting to answer doctors’ questions with evidence	Collection of clinical questions (observation)	Observation and text analysis	103 FPs	Obstacles encountered trying to obtain evidence-based answers to real clinical questions	Clinical questions
Feightner et al 2001 [39]	Explore FPs’ perspectives on how to provide evidence-based preventive clinical practice guidelines to physicians on the internet	Focus groups	Thematic analysis	34 FPs	Preferences for disseminating preventative clinical practice guidelines through the internet	Internet information
González-González et al 2007 [40]	Determine information needs of PCPs and describe their information-seeking patterns	Recording consultations and telephone interview	Classification of questions	112 PCPs; 90 PCPs	Number of questions asked, pursued, and answered; type and topic of questions; time spent pursuing answers; information resources used; perceived barriers to search	Mixed
Heintze et al 2005 [41]	Capture the variety of perceptions and intentions to act and attitudes of GPs regarding their own CME^e^ behavior	Interviews	Thematic synthesis	30 GPs	Perceptions of CME programs	Mixed (CME aspect)
Janes et al 2005 [42]	Investigate health professionals’ attitudes and perceived barriers to using the internet for ongoing professional learning	Cross-sectional study and questionnaire	Inductive approach	175 GPs (survey); 56 GPs (written response)	Barriers to use the internet	Internet information
Lottridge et al 2007 [43]^f^	Investigate differences that impact physicians’ needs of clinical evidence on mobile devices	Interviews	Verbal protocol analysis	47 FPs	Effect of specialty on preferences toward handheld presentation of evidence	Mobile information
Schuers et al 2016 [44]	Explore attitudes and behavior of residents in general medicine and GPs when seeking medical information online	Focus groups	Descriptive analysis	35 GPs	Research topic in general medicine; resource selection; seeking process; research context	Internet information
Vaucher et al 2016 [45]	Assess suggestions of practicing physicians for possible improvements of knowledge transition effectiveness into clinical practice	Interviews; focus groups; online questionnaire	Content analysis; grounded theory	4 GPs (interviews); 25 GPs (focus group); 587 GPs (survey)	Barriers in knowledge transition and suggestions to improve implementation	Mixed
Zack et al 2006 [46]	Better understand GPs’ information needs and preferences to provide basis for developing better information resources	Questionnaire; interview; review medical notes	Grounded theory	47 GPs	Reasons for difficulties and coping strategies; information sources used	Mixed

^a^FP: family physician.

^b^GP: general physician.

^c^N/A: not applicable.

^d^PCP: primary care physician or practitioner.

^e^CME: continuous medical education.

^f^In this study, family physicians did not make up at least 50% of the population. However, the studies compared different groups of doctors and were included in the analysis to evaluate the potential differences between family physicians and other specialties. There was heterogeneity in the studies regarding the term family physician, general practitioner, or primary care practitioner. We decided to use the term of the original study rather than trying to find a common definition of family physicians for different studies. Types of information grouping were performed by medium or source. Due to a lack of one common definition for the information medium or sources among studies, we summarized those studies sharing a comparable or similar definition. However, some studies needed to be grouped by the addressed content rather than the analyzed medium itself.

**Table 2:** Characteristics of quantitative studies.

Author (Reference)	Aims	Data collection	Recruitment and sample size^a^	Main outcomes	Type of information
Andrews et al 2005 [47]	Assess information-seeking behaviors and preferences of clinicians	Cross-sectional survey	Network; PCPs^b^=41	Use of and barriers to information resources	Mixed
Bennett et al 2005 [48]	Assess the way FPs^c^ use the internet to look for clinical information and how patterns vary from other specialists	Fax survey	Fax database; FPs=457	Usefulness of internet as information resource; search pattern compared with other specialties	Internet information
Bernard et al 2012 [49]	Describe characteristics of GPs^d^ using the internet for clinical information search to identify barriers and facilitators to internet use	Cross-sectional survey	Online questionnaire; FPs=721	Internet use for clinical information; obstacles and facilitators for internet use	Internet information
Bjerre et al 2013 [50]	Assess feasibility of using information generated in the context of what could become a “routine” clinical information source	Collection of clinical questions (secondary analysis)	Adjacent study; PCPs=82	Description of type and frequency questions asked (secondary analysis)	Clinical questions
Butzlaff et al 2002 [51]	Demands of GPs toward CME^e^ media, the used CME sources, and their efficacy	Survey	Database; FPs=72	Amount of GPs knowledge acquired after studies. Requirements of GPs toward CME. Sources of GPs’ CME. Efficacy of CME. Significance of the internet.	CME media and internet information
Ciarlo et al 2016 [52]	Identify the specific needs of oncologists and GPs attending cancer patients	Survey	Database; GPs=147	Information sources; questions frequently asked; dealing with uncertainty; satisfaction with information; information seeking and time spent; information needs for specific topics	Mixed
Cogdill et al 2000 [53]	Investigate information needs and information seeking in primary care practices serving as educational sites	Interview with follow-up	University; PCPs=15	Frequencies and categories of questions generated during patient encounter	Clinical questions
Davies 2011 [21]^f^	Determine information needs of physicians	Survey	Professional bodies; PCPs=256	Information needs. Frequency of formulated questions. Use of computers. Preference in locating evidence. Barriers in accessing electronic information.	Electronic information
Denny et al 2015 [54]	Investigate the use of e-resources within the GPs’ education and training sector	Survey	Organizations; GPs=119	Personal and professional characteristics associated with use of e-resources. Preferred sources. Frequency of use. Factors relevant in selection and use.	E-resources
Dwairy et al 2011 [55]	Explore optimal foraging theory to understand information-seeking behavior of GPs, measure costs, and benefits of information-seeking decisions	Logbook and questionnaire	Within region; GPs=115	Time spent on addressing search for clinical information; preferred information source; success in search	Mixed
Ebell et al 2011 [56]	Identify clinical questions health care professionals have and explore whether questions could be used to drive needs assessment for clinical education programs	Collection of clinical questions (observation and survey)	Personal contact and academic; PCPs=25	Description of type and frequency questions asked	Clinical questions
Koller et al 2001 [57]	Clarify reasons for not consulting the internet and identify alternative sources of information for problem solving during patient care	Cross-sectional survey	Database; PCPs=1103	Internet access and use; reasons for not using	Internet information
Kortekaas et al 2015 [58]	Determine how often and how GP trainees search for answers to clinical queries encountered in daily clinical practice	Logs	University; GP trainees=76	Number of clinical queries and answers pursued and retrieved; outcome on decision making; resources used	Mixed
Kostagiolas et al 2015 [59]	Explore the information-seeking behavior of GPs and their attitudes toward participatory medicine	Survey	Organization; GP=174	Information needs and sources; obstacles when seeking information; perception of participatory medicine	Mixed
Kosteniuk et al 2013 [60]	Determine information resources of FPs to update general medical knowledge and make clinical decisions	Cross-sectional survey	Database; FPs=331	Information sources used and found to be most accessible and relevant to needs	Mixed
Kritz et al 2013 [61]^g^	Provide insight on the professional internet use among different subgroups of physicians	Survey	Mixed promotion; GPs=89	Use of online resources; time spent on searches; rate of success; barriers of finding information; search tools	Internet information
Le et al 2016 [62]	Assess GPs’ information-seeking behavior, perceived importance of medical information sources and associations with GPs characteristics	Cross-sectional survey	Organizations; GPs=1580	Frequency of use; perceived importance; associations between GP characteristics and use and importance	Mixed
MacWalter et al 2016 [63]	Determine how GPs use online resources in support of their continuing professional development	Cross-sectional survey	Via email; GPs=383	Internet usage and reasons for use; intergroup comparisons	Internet information
Magin et al 2015 [64]	Establish prevalence and associations of GP trainees in consultation information seeking	Cross-sectional survey	Adjacent study; GP trainees=654	In-consultation information seeking from human or nonhuman source	Mixed
Magin et al 2017 [65]	Establish prevalence and associations of GP trainees generation of learning goals	Cross-sectional survey	Adjacent study; GP trainees=1124	Generation of learning goals	Clinical questions
Magrabi et al 2008 [66]	Determine long-term use of an online evidence system in routine clinical practice	Prospective study (data collection via computer log)	N/A; GPs=59	Usage pattern and user group analysis	Online tool
Ruf et al 2008 [67]	Examine GPs’ attitudes toward and use of the internet and online CME	Survey	Adjacent study; random sample GPs=351	Internet use and importance; frequency and effectiveness of CME	Internet information (CME aspect)
Schwartz et al 2003 [68]	Determine if FP faculty answer their questions using online resources and the proportions of answers that influenced patient care	Prospective study (data collection via computer log)	Practice center; GPs=3	Characteristics of questions generated and answered; search time; resources used; barriers to use	Internet information
Vollmar et al 2008 [69]	Gain understanding of PCPs’ learning media preferences	Survey with follow-up	Postal; PCPs=57	Resources used for CME and effectiveness; demands toward CME media	CME media
Vollmar et al 2009 [70]	Gain understanding of GPs’ preferences for different forms of educational media that will meet CME needs	Survey	Adjacent study; PCPs=264	Internet access and utilization; requirements toward CME media	CME media

^a^Sample size describes family physician staff included. If mixed personnel were surveyed or addressed, nonphysicians were excluded from the description and analysis.

^b^PCP: primary care physician or practitioner.

^c^FP: family physician.

^d^GP: general physician.

^e^CME: continuous medical education.

^f^This survey also included a short literature review but was not excluded from the analysis.

^g^In these studies, family physicians did not make up at least 50% of the population. However, the studies compared different groups of doctors and were included in the analysis to evaluate the potential differences between family physicians and other specialties. There was heterogeneity in the studies regarding the term family physician, general practitioner, or primary care practitioner. We decided to use the term of the original study rather than trying to find a common definition of family physicians for different studies. Types of information grouping were performed by medium or source. Due to a lack of one common definition for the information medium or sources among studies, we summarized those studies sharing a comparable or similar definition. However, some studies needed to be grouped by the addressed content rather than the analyzed medium itself.

#### Type of Information Addressed

Most studies addressed mixed online information sources (n=15) or internet information sources (n=12). Few studies focused on mobile information sources (n=2). One study focused on online information delivered via email (n=1) or online information delivered via an online tool or app (n=1). Some studies focused on the process of generating clinical questions in practice (n=6) or analyzed CME as an online health information resource (n=4).

### Synthesis of Studies

A total of 20 subcategories emerged from the coding of the included studies and were summarized into the following 5 main categories:

1. *Individual needs*: Formed to collect FPs’ expressed individual personal needs, barriers, or demands toward online information before initiating a search. This category collected diverse statements from FPs when they directly identified a personal need or more indirectly explained the individual barriers met.
2. *Access needs*: Formed to collect aspects, needs, or barriers expressed by FPs during the access of online information.
3. *Quality needs*: Formed to collect aspects, needs, or barriers expressed by FPs toward the quality of online information after being accessed.
4. *Utilization needs*: Formed to collect aspects, needs, or barriers regarding the subsequent utilization of the retrieved information.
5. *Implementation needs*: Formed to collect aspects, needs, or barriers regarding consequences and effects that emerged due to or after utilization of online information.

Meta-analyses of survey results were not possible due to the heterogeneity of methods used in data collection. See Table 3 for details of the main categories, items, and item descriptions.

**Table 3:** Main categories, themes, and theme descriptions.

Main category and subcategory		Description		Supporting survey results	
**Individual needs**					
	CME^a^		There is a need for continuous medical education in practice. There is a need for being kept up-to-date [33-36,39,42,44,45].		89.7% ranked knowledge update as high level of importance as information need or motive [59]; 5-point Likert scale (1-2=low, 4-5=high importance), N=174; 80.4% of FPs^b^ use the internet for work-related continuing professional development [63]; survey, N=383.
	Digital skill		There is a lack of internet, computer, or digital skills [31,33,36,39,42,45].		Lack of computer or digital literacy skills ranked low level of importance as an obstacle to obtain information by 70.3% and 65.9% of FPs [59]; 5-point Likert scale (1-2=low, 4-5=high importance), N=174.
	Collaboration		Collaborations among colleagues or with other clinical fields and experts are important [33-36,38,41,42,44-46].		Colleagues as the preferred aid in clinical decision making among FPs [21]; survey, N=256. Colleagues used as information source by 62.4% of the FPs in making clinical decisions [60]; survey; N=330. For learning activities, German FPs use quality circles (75.7%) and colleagues (58.5%) as preferred information source with significant correlation between utilization and efficacy [70]; survey, N=264.
	EBM^c^ skill		There is a lack of methodological and scientific principles regarding the practice of EBM. Methodological and scientific skills regarding EBM are needed [34-36,38,41,44,45].		65.0% of FPs see websites with evidence-based summaries as the leading facilitating factor to use the internet for information seeking in clinical practice [49]; survey, N=721. 45.5% of FPs see difficulties in quality appraisal as a leading barrier to using the internet for CME [67]; survey, N=349.
	Prefer analogue		Analogue information may be preferred over electronic resources by FPs [35,39,42,45].		Medical textbooks (66.4%) or books or printed journals (86.3%) used by FPs to make specific clinical decisions regarding patient care [49,60]; surveys, N=721 and 330, respectively.
**Access needs**					
	Time		Time to look up or access information is missing. Information access should be quick [21,31,34,35,38-42,44,45].		Lack of time ranked as a leading important obstacle when seeking information [59]; 65.3% high importance, 5-point Likert scale (1-2=low, 4-5=high importance), N=174. 47.0% of FPs ranked lack of time as second most important barrier when searching the internet for clinical information [49]; survey, N=721. FPs spent least amount of time in complex queries compared with other specialties [61]; survey, N=500. Time to search was ranked as the most frequent barrier to look for information [21]; survey, N=256.
	Simple		Online resources should facilitate easy access to information. Navigational aspects are important for access to information [32,36,39,42-46].		FP registrars named ease of navigation as a factor relevant to use of e-resources [54]; mean 4.32, SD 0.61, 5-point Likert scale (1=not important, 5=very important), N=119. 61.3% of FPs ranked navigation difficulties first as physician internet barrier [48]; survey, N=457.
	Cost		Access to information is expensive. Access to information should be free [32,41,42,44,45].		Cost was ranked as the second highest obstacle when seeking information [59]; 59.2% high importance, 5-point Likert scale (1-2=low, 4-5=high importance), N=174.
	Language		Foreign language can be a barrier in the information-seeking process [44].		Language barrier was ranked third by 34.1% of FPs as an obstacle when seeking information in clinical practice [49]; survey, N=721.
	Technical		Hardware, software, or technical issues prevent access to information [32,38,39,42].		Most reported difficulties when using online resources for professional development: 62.7% slow internet connection; 49.9% additional software needed; 46.2% access to website restricted; 42.6% problems logging into online resource; and 37.3% internet connection problems other than speed [63]; survey, N=383.
**Quality needs**					
	Credible		Information and the institution offering it should be credible, transparent, and trustworthy. Information should be independent from pharmaceutical firms or industry [32,34-36,38,39,41,44-46].		Reliability is the second most favored attribute regarding tools for CME [70]; 89.8% very important, 3-point ordinal scale (0=unimportant, 2=very important), survey N=264. Pharmaceutical sales representatives are the least used information source by FPs [60]; 4.2%, survey, N=330.
	Concise		Overabundance of information can result in an ineffective search of information. Information should be preselected and comprehensive to FPs’ relevant topics [31,34,35,38,39,41,42,44-46]. FPs need short and concise summaries of information [35,38,39,41,43-46].		“Too much information to scan” named as leading barrier (47.7%) to internet use for information seeking and identified “evidence-based summaries” and “selected documents” as leading facilitating factors for information seeking (65.0% and 54.4%, respectively) [49]; survey, N=721. “Content filters” perceived as an important tool for information search, identified by 48.0% of FPs [61]; survey; N=89.
	Up-to-date		Information should be recent and up-to-date [35,38,39,42,45,46].		“Creation date listed” was identified as an important factor relevant to GPs’^d^ use of e-resources [54]; mean 4.22, SD 0.72, 5-point Likert scale (1=not important, 5=very important) survey, N=119.
	Specific		There is a need for specific and in-depth information among FPs that is highly variable and dependent on the situation (eg, rare diseases and pediatric doses) [32,34-36,38,39,41,44,46].		66.7% of FPs search for specific patient information, 44.0% of FPs identified the lack of availability of specific information as a barrier to using the internet [48]; survey, N=457.
**Utilization needs**					
	Usability		FPs identify easy navigation and organized content as important for the daily usability of an electronic resource [32,35,38,39,43,46].		—^e^
	Science-practice gap		FPs note an existing gap between scientific literature and the questions arising from daily practice [38,45].		—
	Doctor-patient-relationship		FPs see implications for the doctor-patient relationship, when information search is done during the patient encounter [33,35,44].		71.0% of FPs name “disturbance of doctor-patient-communication” as a leading reason for not using the internet [57]; survey, N=1103.
**Implication needs**					
	Relevancy for daily practice		Electronic information should be useful or relevant to daily practice and individual setting. Information should aid or improve the process of clinical decision making [32,34,35,38,41,42,44-46].		“Relevant to practice” is rated as a very important requirement and most favored attribute of educational media use by 93.3% of FPs [70]; 3-point ordinal scale (0=unimportant, 2=very important), survey N=264. 27.0% of FPs name “low relevance for clinical practice” as a barrier to using the internet for information seeking, and nearly half of the FPs see more relevancy for clinical practice as a facilitating factor [49]; survey; N=721.
	Patient education		Information should be useful for patient education [32,34-36,39,43,44,46].		93.5% of FPs use the internet for obtaining information to give to a patient [63]; survey, N=383.
	Justification of practice		FPs search for information to justify practice or clinical decision [34-36,39,41,44,46].		Improvement of clinical decision making and confirmation of decision are among the most frequently named impacts of information search among FP trainees [58]; 25.8% and 22.7% of clinical queries in daily practice; survey; N=76.

^a^CME: continuous medical education.

^b^FP: family physician.

^c^EBM: evidence-based medicine.

^d^GP: general physician.

^e^No substantiating quantitative results are displayed.

#### Individual Needs

#### CME

FPs identified a need for CME in everyday practice [33-36,39,42,44,45] but did not rank the internet as the most preferred source [36,49] for obtaining CME-related information. Although the work-related utilization of the internet for CME is quite high [63], FPs appear to prefer personal medical education such as colleagues and quality circles for updating their knowledge [51,67,70].

#### Digital Skill

FPs mentioned a lack of digital, computer, or internet skills as a potential barrier in obtaining online health information [31,33,36,39,42,45]. However, the lack of digital or technical skills was not mentioned as a leading obstacle to obtaining online information [48,49,57,59,67]. A cross-sectional survey made more precise distinctions in mentioning digital or technical difficulties when using online resources, displaying the variety of digital or technical barriers that can occur when using new technologies (eg, log-in problems and need for additional software) [63].

#### Collaboration

FPs expressed the need for collaborations with colleagues or experts throughout different disciplines and institutions (practice, hospital, and universities) when seeking information [33-36,38,41,42,44-46]. Quantitative data from surveys support the utilization of colleagues and experts as an important information resource for FPs [21,51,52,55,57-60,62,69,70]. Colleagues were the resource with the highest success rate when obtaining information among FPs, being more efficient than search engines or websites [55]. Young FP registrars named face-to-face contact with educators or colleagues as the second most preferred resource after using e-resources [54].

#### Evidence-Based Medicine Skill

FPs realized a lack of various skills and competences relating to methods and principles of practicing evidence-based medicine (EBM; eg, literature search and critical appraisal) [34-36,38,41,44,45]. Surveys mentioned the difficulty in obtaining quality appraisals as a hindrance to their use of the internet for CME [67] and identified websites with evidence-based summaries as facilitators of their use of the internet for information seeking in clinical practice [49].

#### Prefer Analogue

In a few qualitative studies, analogue sources of information were preferred by some FPs over electronic resources [35,39,42,45]. Quantitative studies show varying and inconclusive results concerning the FPs’ most preferred sources of information [21,47,51,52,55,58-60,62,67,69,70].

#### Access Needs

#### Time

Lack of time was frequently referred to as a barrier to accessing information. Quick access to information was demanded by FPs in both qualitative studies and surveys [21,31,34,35,38-42,44,45,49,51,57,59,69,70]. FPs were reported as devoting the least amount of time to complex queries, and they are more likely to perceive a lack of time than other specialists [61]. FPs spent 18 min on average on their searches for clinical information [55]. FPs refer to the lack of time as a leading barrier to obtaining information from the internet [49,57,59]. FPs also ranked the attribute *fast* as a leading criterion for the efficient utilization of information [51,69,70].

#### Simple

FPs mentioned easy access as an important requirement in the process of seeking and obtaining information. Emphasis was laid on simple technological aspects or technological tools that enhanced information access [32,36,39,42-46]. Surveys supported the fact that complex technological procedures appear to be a hindrance to the access of online information [49,63,67]. User friendliness was mentioned as an important requirement in obtaining electronic information [51,69,70]. Another aspect was the identification of navigation difficulties as a barrier to obtaining information from the internet [48] as well as mentioning the ease of navigation as a factor that was highly relevant to FPs when using e-resources [54].

#### Cost

FPs named costs as a barrier to accessing information. On the one hand, they expressed the need for free access to information, yet on the other hand, they mentioned costs of hardware and software as a hindrance to obtaining information [32,41,42,44,45]. Surveys supported cost and cost-effectiveness as a factor for FPs when using CME [51,62,67,69,70], although no obvious conclusion was drawn from the importance of this factor as a barrier to accessing information in general [49,59].

#### Language

Qualitative studies rarely mentioned languages as an obstacle for obtaining information [44]. However, surveys identified foreign languages as a possible barrier in the process of seeking information [49,59]. For German FPs, language is of medium importance when using the internet [67,69,70].

#### Technical

The technical aspects identified as preventing the access of information or displaying a barrier to the process of seeking information were named in several studies covering a wide variety of technical, hardware and software, or internet-related problems [32,38,39,42]. A quantitative survey among Scottish FPs identified several distinct issues, such as a slow connection or incompatible software, when accessing information for CME [63].

#### Quality Needs

#### Credible

FPs’ needs regarding the quality of information, trustworthiness, credibility, and transparency of information and the institution generating this information were frequently named in qualitative studies [34-36,38,39,44-46]. Transparency and credibility were often linked to the need for information to be independent from the pharmaceutical industry [32,38,41,44,45]. Quantitative studies supported the need for trustworthy, credible, and transparent information among FPs [48,51,57,60,63,67,69,70].

#### Concise

FPs cited an overabundance of information as a barrier to the process of searching for specific or relevant information. The internet and other electronic information resources were perceived as containing an untamed *information jungle*, hindering the effectiveness of researching FP-relevant information [31,34,35,38,39,41,42,44-46]. This result was supported by several surveys that addressed the need for concise information or identified *too much* or *confusing* information as an access barrier [49,51,54,57,59,61,67,69,70]. Another aspect identified by several qualitative studies was the FPs’ need for short and concise summaries of information [35,38,39,41,43-46].

#### Up-to-Date

Another need identified by FPs was the currency of information available [35,38,39,42,45,46], which was chiefly cited by qualitative analyses rather than quantitative studies [54,67].

#### Specific

FPs seemed to show differing needs for specific information depending on the particular clinical question at hand and the individual patient situation. These needs could cover anything from detailed pediatric drug dosing to diagnostic criteria for rare diseases, but could not be narrowed down to any specific or homogenous topic [32,34-36,38,39,41,44,46]. Therefore, quantitative data were too heterogeneous to present a distinct pattern of those specific needs emerging from surveys. However, a survey listed the unavailability of specific information as a barrier for FPs [48]. It was not an aim of this review to analyze the distinct medical information FPs were searching for, but some of the included studies identified these topics or developed or used the taxonomy of clinically generated questions by FPs [37,40,50,56,64,65,68]. This supported the highly heterogeneous field of clinical questions that could arise from the FPs’ daily routine. Cook et al [35] noted that the complexity of clinical questions was an important aspect to consider among FPs.

#### Utilization Needs

#### Usability

The most prominent aspect retrieved was the need for easy navigation and an organized display of structured content [32,35,38,39,43,46]. Minor aspects retrieved from some studies also mentioned the need for short and summarized information [35,39] and aspects regarding mobile or tablet resources such as physical size, screen requirements, or applications used [32,43,45]. Quantitative evidence identifying needs as suitable for daily practice utilization was sparse [70].

#### Science-Practice Gap

Few qualitative studies mentioned that scientific literature failed to address and reflect on the relevant problems emerging from daily practice, omitting the connection of academic centers to daily practice [38,45]. None of the included surveys directly measured this aspect. Few surveys report that FPs perceived a lack of specific information [48] or the low relevance to clinical practice [49] as a barrier to searching for information on the internet.

#### Doctor-Patient Relationship

Qualitative studies suggested considering the setting of the FP encounter with the patient, and possible positive and negative implications on the doctor-patient relationship, as a consideration when information searches were conducted during the encounter [33,35,44]. One focus group study, in particular, named the complexity of questions that arose in general practice as a barrier to searching for information [35]. An older survey of Swiss doctors identified the interruption of doctor-patient communication as a reason for not using the internet [57].

#### Implication Needs

#### Relevance for Daily Practice

One important implication for the FPs’ everyday practice was a reported lack of usefulness and relevance of electronic resources in the daily clinical routine. FPs noted that information should be applicable to their specific daily situations, rather than general guidelines and recommendations [32,34,35,38,41,42,44-46]. Surveys supported the need for information relevance to daily practice [51,67,69,70] or identified low relevance as a barrier to information seeking [49]. Furthermore, surveys reported the need for information to make improved clinical decisions [58,63].

#### Patient Education

An important viewpoint of many FPs was the usefulness of retrieved information for patient education or the need for information supporting the patients’ involvement in the process of explanation, identified by a number of qualitative studies [32,34-36,39,43,44,46]. A survey of Scottish FPs reported that over 90% of them used the internet to obtain information for the patient [63] or advise patients on internet health resources [47].

#### Justification of Practice

Qualitative studies also showed that general physicians (GPs) searched and used information in everyday practice to reaffirm preexisting knowledge or to justify their clinical decisions [34-36,39,41,44,46]. The search strategies of FP trainees also demonstrated the impact on clinical decision making or the confirmation of a diagnosis [58]. Surveys that developed or relied on the taxonomy arising from FPs’ daily practice also supported this finding. The most common question types could be classified according to the categories *diagnosis* and *treatment* [37,40,50,56,64,65,68]. Surveys also show that the topics *diagnosis* and *treatment* were important information-seeking motives or information needs among FPs [52,53,59].

## Discussion

### Principal Findings

This study presented 5 main aspects of FPs’ needs toward online health information:

1. Several individual needs exist for FPs before online information is accessed, such as the need for digital and EBM skills, preference for analogue information or a desire for CME, and the need for interspecialist collaborations.
2. Needs that are connected with the access of online information, such as simple access, technical barriers, a good cost-benefit ratio, or suitable languages.
3. Needs that address aspects of quality itself, for instance, credible and recent information. The most interesting aspects of quality revealed a converse need for concise information, on the one hand, as well as the need for specific in-depth information, on the other hand.
4. Needs that are concerned with the feasible utilization of obtained information, such as the suitability of information to the distinct and unique situations in family practice.
5. Needs that reflect the subsequent implications of using information that is tailored to FP practice, clinical judgment and decision making, and patient education as well as providing additional value to the FPs' future practice.

### Comparison With Prior Work

The impact of the internet on the information needs of primary care was reviewed in 1999 and identified FPs’ need to manage information overload as well as the need for specific and simple information [5]. This study confirms these findings within the quality needs category and thus confirms prior work as still valid. Rural health professionals have information needs directly relating to patient care and therapy, and they cited a lack of time or technological literacy as barriers to obtaining information [6]. Our study supports the relevance of patient-related needs mainly in the utilization and implication needs category. It also confirms time and technological aspects in the access needs category.

Dawes and Sampson [7] noted heterogeneous information-seeking behavior among doctors in 2003 and asked for careful planning in delivering useful, relevant, and fast information to physicians, supported by our findings within the main categories of utilization and implication needs. A noteworthy review from 2006 identified the information-seeking obstacles to primary care physicians in the context of established EBM processes [8]: (1) acknowledging an information gap, (2) formulating a question, (3) seeking relevant information, (4) formulating an answer, and (5) applying the answer to patient care. Although our review is able to confirm most of the barriers reported by Coumo and Meijman [8], it intends to present an adapted classification of the steps necessary for FPs to obtain such information.

The FPs’ information needs cannot be completely met by only providing high-quality information through newly tailored online sources. New content, new technologies, or new systems must address seeking competencies, strategies of utilization, and the implications generated in family practice, as our results revealed. The internet’s role in needs, the information-seeking patterns, and the sources utilized was partly reviewed by Davies [9]. In agreement with some of our subcategories, prominent barriers identified in information searches were lack of time, lack of information technology skills, and lack of search skills, although needs were often related to diagnosis and therapy. Physicians’ information needs are often related to diagnosis, therapy, and patient care, as confirmed by literature reviews from 2010 and 2013 [10,11]. The most recent systematic review in this field of work was conducted by Del Fiol et al [12] in 2014, confirming that clinicians raise questions about patient care. Although our study confirms the findings of these more recent reviews, none of them have been exclusively focused on FPs. Therefore, a substantiating comparison remains complex.

### What Is New and Where to Go From Here?

Despite confirming prior work, this study seeks to highlight possible future work emerging from the results presented. The main categories and subcategories indicate that needs toward online information by FPs seem to be closely associated with CME and EBM. Evidence suggests that EBM interventions improve short-term knowledge, but there is little evidence of a change in long-term knowledge, attitudes, or clinical practice [77]. Despite technological advancements, half of the clinical questions still seem to be unanswered at the point of care [12]. No study has directly measured the effects of these interventions on patients’ outcomes or FPs’ behavior [8]. The inability to search for the literature and critically appraise the content—both inevitable steps of EBM [78]—were identified as barriers to obtaining information in the first place by this study. We suggest that future work should focus on these intermingling aspects of information need, CME, EBM, and daily routine in the primary care setting. It should not abandon the implications and effects on FPs’ behavior or patient outcomes that occur after an information search or when a question is not pursued.

Searching for and critically appraising primary literature in a short amount of time remains a major obstacle in primary care, urging FPs to express needs for concise secondary, credible, free, and simple information that also provides valuable and specific medical information. This converse need for short and concise, but also in-depth, information for an FP, in our opinion, has neither been met by new online information platforms nor by science contributing to the information translation with relevant research into the FPs’ daily practice. An FP’s need for information rarely starts with a scientific definition of an illness or an update on epidemiology, but with a specific question on individual patients and with direct impact on the situation presented during the consultation. The vast amount of information available across multiple platforms and sources emerges as an obstacle to both initiating and pursuing a clinical question in the FPs’ daily practice and consultation. Lack of time remains a major obstacle to information retrieval among FPs, despite the abundance of online information. This emphasizes the fact that online information has not yet fully evolved to satisfy the needs of FPs, explaining that FPs may still prefer colleagues and analogue information in many situations over digital solutions, as it is free, delivered by specialist colleagues, fast, simple, and concise.

### Methodological Strengths and Limitations

To our knowledge, this is the first systematic review that analyses the available qualitative and quantitative evidence focused solely on FPs using online health information. As our search was not limited to a specific study design, we feel it is unlikely, but not impossible, that further relevant publications are available. However, the heterogeneity among countries and their unique health care systems made it challenging to find a common term for *family physician*, *family practitioner*, *general practitioner,* and *primary care physician* among the studies included. Both the differences in health care and educational systems can result in a heterogeneous study population of *family physicians*. As this study excluded works from countries with a completely different primary care or health care system or far less developed technological infrastructure than the majority of those in the included studies, this review may display a bias in this aspect of selection.

There is no established methodological approach for synthesizing both qualitative and quantitative data [79], and a variety of methods seems plausible [74]. We, therefore, used specific steps for quality appraisal and synthesis of the studies by following the thematic synthesis by Thomas and Harden [71], referring to the study by Möhler and Meyer [72], and applying the well-established (eg, [28-30]) critical appraisal tools CASP [26] and AXIS [27]. Despite independent review from 2 scientists, the critical appraisal and the reported items cannot cover all aspects of the heterogeneous body of evidence. We neither wanted to unduly appraise nor indecently criticize the studies’ quality or the authors’ contribution to the scientific community. The final critical appraisal must remain with the scientist using the included original study. According to Hong et al [74], when addressing one overall review question, as is the case with this review, a sequential study synthesis design is applicable. Despite presenting qualitative and quantitative results parallel to our results, we first synthesized qualitative themes and then collected evidence from quantitative studies to support and enrich these developed themes.

We tried to minimize an aspect we named *technological bias* by limiting studies from the years spanning 2000 to 2020. Through the chosen search terms as well as the established exclusion criteria, we sought to ensure that only studies regarding electronic information were included, when the internet and computers were broadly available in most countries. Still, the technical developments of the last 20 years have been expeditious and have resulted in a rapidly developing infrastructure, hardware, and software environment. We noticed the resulting variety of electronic information, ranging from CD-ROM to very recent online databases. Therefore, a small technological bias remains, especially due to older studies that analyzed technological information systems and corresponding seeking behaviors, which are generally no longer used or even obsolete in 2020 (eg, CD-ROM and Palm OS).

### Conclusions: FPs’ View

Although technology and infrastructure, methods, and sources of information retrieval have changed, the needs and barriers of FPs to information seeking and retrieval have not. The question arises, why do technological advancements not succeed in fulfilling the information needs of FPs?

We propose the following two main answers to this question:

1. Human sources of information, such as colleagues, play an important role. FPs are the center of an afferent information flow, as they receive health information from hospitals and other specialists. The FP provides primary care for patients presenting with a variety of illnesses and questions. There is a tension field for the FPs as information givers with an efferent information flow toward the patient. FPs need to develop coping strategies to tackle the demands met in this center of bidirectional information flow by seeking CME and EBM, both instruments to improve knowledge and information retrieval.
2. FPs acknowledge their need for digital skills to search and find the information needed in the online information jungle. It is interesting to note that the methods used for providing this information have come to signify the transition from the analogue to the digital era, although the way of presenting this information for the FPs’ daily work has not yet kept up with this transition.

This review aims to contribute to a (1) FP-specific and (2) an updated systematic body of research that also sought to analyze (3) the influencing factors affecting needs and requirements for online information in primary care. This study concludes that FPs show specific needs for online information due to their daily routine and broad working environment. Future information resources, whether online or analogue, must address the needs emerging from the primary care setting as well as rethink the way in which information is adapted to the needs of the digital age. This requires not only the development and implementation of new information systems but also the evaluation of their effects on both physicians and patients. Finally, science should also rethink the way online medical information is disseminated, adapted, and translated into daily practice.

## Appendix

Multimedia Appendix 1 Search terms and search characteristics.

Multimedia Appendix 2 Methodological quality of qualitative and mixed methods studies.

Multimedia Appendix 3 Methodological quality of quantitative studies.

## Abbreviations

AXIS: appraisal tool for cross-sectional studies
CASP: critical appraisal skills program
CME: continuous medical education
EBM: evidence-based medicine
FP: family physician
GP: general physician
MeSH: Medical Subject Headings
