# Supplementary material for: The Online Health Information Needs of Family Physicians: Systematic Review of Qualitative and Quantitative Studies
Source: J Med Internet Res. 2020 Dec 30;22(12):e18816. doi: 10.2196/18816 (PMC7806443; doi:10.2196/18816)
Supplement: Multimedia Appendix 1 [file jmir_v22i12e18816_app1.docx]

## Multimedia Appendix

### Multimedia Appendix 1: Search Terms and Search characteristics

| **MEDLINE via PubMed** |
| --- |
| #1 general practitioner* ti,ab |
| #2 family practitioner* ti, ab |
| #3 family doctor* ti,ab |
| #4 family physician* ti,ab |
| #5 general practice **AND** physician ti,ab |
| #6 primary care **AND** physician ti,ab |
| #7 “General Practitioners” [MeSH] |
| #8 “Physician, Primary Care” [MeSH] |
| #9 **#1 OR #2 OR #3 OR #4 OR #5 OR #6 OR #7 OR #8** |
| #10 health information ti,ab |
| #11 medical information ti,ab |
| #12 treatment information ti,ab |
| #13 healthcare information ti,ab |
| #14 information seeking behavior ti ab |
| #15 “Health Information Systems” [MeSH] |
| #16 “Online Systems” [MeSH] |
| #17 “Information Services [MeSH] |
| #18 “Education, Continuing, Medical” [MeSH] |
| #19 “Information Seeking Behavior” [MeSH] |
| #20 **#10 OR #11 OR #12 OR #13 OR #14 OR #15 OR #16 OR #17 OR #18 OR# 19** |
| #21 expectation* ti,ab |
| #22 usage ti,ab |
| #23 utilization ti,ab |
| #24 behavi* ti,ab |
| #25 needs ti,ab |
| #26 demands ti,ab |
| #27 require* ti,ab |
| #28 perception* ti,ab |
| #29 barrier* ti,ab |
| #30 “Needs Assessment” [MeSH] |
| #31 “Attitude of Health Personel” [MeSH] |
| #32 **#21 OR #22 OR #23 OR #24 OR #25 OR #26 OR #27 OR #28 OR #29 OR #30 OR #31** |
| #33 **#9 AND #20 AND #32** |
| #34 limit #33 to yr=”2000-2020” |
| Notes:  date searched: initial 2 May 2018, updated 21 January 2020  language: limit to English AND German |
| **Web of Science** |
| #1 TS=general practitioner* |
| #2 TS=family practitioner* |
| #3 TS=family doctor* |
| #4 TS=family physician* |
| #5 TS=primary care AND physician* |
| #6 TS=general practice AND physician* |
| #7 #**1 OR #2 OR #3 OR #4 OR #5 OR #6** |
| #8 TS=”health information” |
| #9 TS=”medical information” |
| #10 TS=”treatment **AND** information” |
| #11 TS=”healthcare information” |
| #12 #**8 OR #9 OR #10 OR #11** |
| #13 TS=expectation* |
| #14 TS=usage |
| #15 TS=utilization |
| #16 TS=behavi* |
| #17 TS=needs |
| #18 TS=demands |
| #19 TS=require* |
| #20 TS=perception* |
| #21 TS=barrier* |
| #22 #**13 OR #14 OR #15 OR #16 OR #17 OR #18 OR #19 OR #20 OR #21** |
| #23 #**7 AND #12 AND #22** |
| #24 timespan #22 “2000-2020” |
| Notes:  date searched: initial 2 May 2018, updated 21 January 2020 Index: SCI-EXPANDED language: limit to English AND German |
| **Scopus** |
| #1 TITLE-ABS-KEY=”general practitioner*” |
| #2 TITLE-ABS-KEY=”family practitioner*” |
| #3 TITLE-ABS-KEY=”family doctor*” |
| #4 TITLE-ABS-KEY=”family physician*” |
| #5 TITLE-ABS-KEY=”primary care AND physician*” |
| #6 TITLE-ABS-KEY=”general practice AND physician*” |
| #7 **#1 OR #2 OR #3 OR #4 OR #5 OR #6** |
| #8 TITLE-ABS-KEY=””health information” |
| #9 TITLE-ABS-KEY=”medical information” |
| #10 TITLE-ABS-KEY=”treatment information” |
| #11 TITLE-ABS-KEY=”healthcare information” |
| #12 TITLE-ABS-KEY=”information seeking behave*” |
| #13 **#8 OR #9 OR #10 #11 OR #12** |
| #14 TITLE-ABS-KEY=”expectation*” |
| #15 TITLE-ABS-KEY=”usage” |
| #16 TITLE-ABS-KEY=”utilization” |
| #17 TITLE-ABS-KEY=”behavi*” |
| #18 TITLE-ABS-KEY=”needs” |
| #19 TITLE-ABS-KEY=”demands” |
| #20 TITLE-ABS-KEY=” require*” |
| #21 TITLE-ABS-KEY=”perception*” |
| #22 TITLE-ABS-KEY=”barrier*” |
| #23 **#14 OR #15 OR #16 OR #17 OR #18 OR #19 OR #20 OR #21 OR #22** |
| #24 **#7 AND #13 AND #23** |
| #25 LIMIT #24 TO (LANGUAGE ”English, German” |
| #26 LIMIT #24 TO (PUBYEAR, 2000 to 2020) |
| Notes:  date searched: initial 2 May 2018, updated 21 January 2020 |
