# Supplementary material for: The Online Health Information Needs of Family Physicians: Systematic Review of Qualitative and Quantitative Studies
Source: J Med Internet Res. 2020 Dec 30;22(12):e18816. doi: 10.2196/18816 (PMC7806443; doi:10.2196/18816)
Supplement: Multimedia Appendix 2 [file jmir_v22i12e18816_app2.doc]

### Multimedia Appendix 2: Methodological quality of qualitative and mixed-methods studies.

|  | Badran et al 2015 [37] | Barrett et al 2004 [38] | Boissin 2005 [39] | Bryant 2004 [40] | Cook et al 2013 [41] | Cullen 2002 [42] | Ely et al 2000 [43] | Ely et al 2002 [44] | Feightner et al 2001 [45] | González-González et al 2007 [46] |
| --- | --- | --- | --- | --- | --- | --- | --- | --- | --- | --- |
| Was there a clear statement of the aims of the research? | Y | N | Y | Y | Y | Y | Y | Y | Y | Y |
| Is a qualitative methodology appropriate? | Y | Y | Y | Y | Y | Y | Y | Y | Y | Y |
| Was the research design appropriate to address the aims of the research? | Y | Y | Y | Y | Y | Y | Y | Y | Y | Y |
| Was the recruitment strategy appropriate to the aims of the research? | Y | N | N/A | N/A | Y | Y | Y | Y | Y | Y |
| Was the data collected in a way that addressed the research issue? | Y | N/A | N/A | Y | Y | Y | Y | Y | Y | Y |
| Has the relationship between researcher and participants been considered? | Y | N | N | N | N | N | N | Y | N | N |
| Have ethical issue been taken into consideration? | Y | N | N | N | N | N | N | N | Y | N |
| Was the data analysis sufficiently rigorous? | Y | Y | N | Y | Y | Y | Y | Y | Y | N |
| Is there a clear statement of findings? | Y | Y | Y | Y | Y | Y | Y | Y | Y | Y |
| How valuable is the research? | + | + | + | + | + | + | + | + | + | + |

|  | Heintze et al 2005 [47] | Janes et al 2005 [48] | Lottridge et al 2007 [49] | Schuers et al 2016 [50] | Vaucher et al 2016 [51] | Zack et al 2006 [52] |
| --- | --- | --- | --- | --- | --- | --- |
| Was there a clear statement of the aims of the research? | Y | Y | Y | Y | Y | Y |
| Is a qualitative methodology appropriate? | Y | Y | Y | Y | Y | Y |
| Was the research design appropriate to address the aims of the research? | Y | Y | Y | Y | Y | Y |
| Was the recruitment strategy appropriate to the aims of the research? | Y | Y | Y | Y | Y | Y |
| Was the data collected in a way that addressed the research issue? | N | N | Y | Y | N/A | Y |
| Has the relationship between researcher and participants been considered? | N | N | N | Y | Y | N |
| Have ethical issue been taken into consideration? | N | Y | Y | Y | Y | Y |
| Was the data analysis sufficiently rigorous? | Y | Y | Y | Y | Y | Y |
| Is there a clear statement of findings? | Y | Y | Y | Y | Y | Y |
| How valuable is the research? | + | + | + | + | + | + |

Notes: Symbols indicate: N = No; Y =Yes + = valuable, N/A = not applicable, no comment
