# Supplementary material for: The Online Health Information Needs of Family Physicians: Systematic Review of Qualitative and Quantitative Studies
Source: J Med Internet Res. 2020 Dec 30;22(12):e18816. doi: 10.2196/18816 (PMC7806443; doi:10.2196/18816)
Supplement: Multimedia Appendix 3 [file jmir_v22i12e18816_app3.doc]

### Multimedia Appendix 3: Methodological quality of quantitative studies.

|  | Andrews et al  2005 [53] | | Barrett et al 2004* [38] | | | | Bennett et al 2005 [54] | | Bernard et al 2012 [55] | | | Bjerre et al 2013 [56] | Bryant 2004* [40] | | | Butzlaff et al 2002 [57] | | | Ciarloet al 2016 [58] | | | Cogdill et al 2000 [59] | | | | Cullen 2002* [42] | | | Davies 2011 [21] | | | Denny et al 2015 [60] | | | Dwairy et al 2011 [61] | | |  |
| --- | --- | --- | --- | --- | --- | --- | --- | --- | --- | --- | --- | --- | --- | --- | --- | --- | --- | --- | --- | --- | --- | --- | --- | --- | --- | --- | --- | --- | --- | --- | --- | --- | --- | --- | --- | --- | --- | --- |
| Were the aims of the study clear? | Y | | | N | | | Y | | Y | | | Y | Y | | | Y | | | Y | | | | Y | | | Y | | | Y | | | Y | | | | Y |  | |
| Was the design appropriate for the stated aims? | N | | | Y | | | Y | | Y | | | Y | Y | | | N | | | Y | | | | Y | | | Y | | | N | | | N | | | | Y |  | |
| Was the sample size justified? | Y | | | N | | | Y | | Y | | | N/A | N | | | N | | | Y | | | | N | | | Y | | | N | | | N | | | | N/A |  | |
| Was the target/reference population clearly defined? | N | | | Y | | | Y | | Y | | | Y | Y | | | Y | | | N | | | | N | | | Y | | | N | | | Y | | | | Y |  | |
| Was the sample taken from an appropriate population base? | Y | | | Y | | | Y | | Y | | | N/A | N | | | Y | | | N | | | | N | | | Y | | | Y | | | N/A | | | | Y |  | |
| Was the selection process likely to select subjects that were representative? | Y | | | N/A | | | Y | | N | | | N/A | N | | | Y | | | N | | | | N | | | Y | | | N | | | N | | | | Y |  | |
| Were measures undertaken to address and categorize non-responders? | N | | | N | | | N | | N | | | N | N | | | N | | | N | | | | N | | | N | | | N | | | N | | | | N |  | |
| Were the risk factor and outcome variables measured appropriate to the aims of the study? | N/A | | | Y | | | Y | | Y | | | Y | Y | | | Y | | | Y | | | | Y | | | Y | | | Y | | | Y | | | | Y |  | |
| Were the risk factor and outcome variables measured correctly using instruments that had been trailed/piloted or published previously? | N | | | N | | | Y | | N/A | | | Y | N | | | N | | | N | | | | N | | | N | | | Y | | | N | | | | Y |  | |
| Is it clear what was used to determine statistical significance and/or precision estimate? | N/A | | | N/A | | | Y | | Y | | | Y | N | | | Y | | | Y | | | | N | | | N | | | Y | | | Y | | | | Y |  | |
| Were methods sufficiently described to enable them to be repeated? | Y | | | Y | | | Y | | Y | | | Y | N | | | Y | | | Y | | | | N | | | Y | | | Y | | | Y | | | | Y |  | |
| Were the basic data described adequately? | Y | | | Y | | | Y | | Y | | | Y | N | | | Y | | | Y | | | | N | | | Y | | | Y | | | Y | | | | Y |  | |
| Does the response rate raise concerns about non-response-bias? | N | | | N | | | N | | N/A | | | N/A | N | | | N | | | N/A | | | | N/A | | | N | | | N/A | | | N/A | | | | N |  | |
| Was information on non-responders described? | N | | | N | | | N | | N | | | N | N | | | N | | | N | | | | N | | | N | | | N | | | N | | | | N |  | |
| Were the results internally consistent? | N/A | | | N/A | | | N/A | | N/A | | | Y | N/A | | | N/A | | | N/A | | | | N/A | | | N/A | | | N/A | | | N/A | | | | N/A |  | |
| Were the results for analyses, described in the methods, presented? | N/A | | | N/A | | | N/A | | Y | | | Y | N | | | Y | | | Y | | | | Y | | | Y | | | Y | | | Y | | | | Y |  | |
| Were the authors’ discussions and conclusions justified by the results? | Y | | | Y | | | Y | | Y | | | Y | Y | | | Y | | | Y | | | | Y | | | Y | | | Y | | | Y | | | | Y |  | |
| Were the limitations of the study discussed? | N | | | N | | | N | | Y | | | Y | Y | | | Y | | | Y | | | | N | | | N | | | Y | | | Y | | | | Y |  | |
| Were there any funding sources or COI? | N | | | N | | | Y | | Y | | | Y | N/A | | | N | | | Y | | | | Y | | | N | | | N | | | Y | | | | Y |  | |
| Was ethical approval or consent of participants attained? | N | | | N | | | N | | Y | | | Y | N | | | N | | | Y | | | | N | | | N | | | Y | | | Y | | | | Y |  | |
|  | Ebell et al 2011 [62] | González-González et al 2007* [46] | | | | Koller et al 2001 [63] | | | Korte-kaas et al 2015 [64] | | Kostagiolas et al 2015 [65] | | | | Kosteniuk et al 2013 [66] | | | Kritz et al2013 [67] | | | Le et al 2016 [68] | | | MacWalter et al 2016 [69] | | | | Magin et al 2015 [70] | | | Magin et al 2017 [71] | | | Magrabiet al 2008 [72] | | | | |
| Were the aims of the study clear? | Y | Y | | | Y | | | Y | | Y | | | | Y | | | Y | | | Y | | | | | Y | | Y | | | Y | | | N | | | | |  |
| Was the design appropriate for the stated aims? | Y | Y | | | Y | | | Y | | Y | | | | Y | | | Y | | | Y | | | | | Y | | Y | | | Y | | | Y | | | | |  |
| Was the sample size justified? | N | Y | | | N | | | N | | N | | | | Y | | | N/A | | | N/A | | | | | N | | Y | | | Y | | | N | | | | |  |
| Was the target/reference population clearly defined? | N | Y | | | Y | | | Y | | Y | | | | Y | | | Y | | | Y | | | | | Y | | Y | | | Y | | | N | | | | |  |
| Was the sample taken from an appropriate population base? | N/A | Y | | | Y | | | Y | | Y | | | | Y | | | Y | | | Y | | | | | Y | | Y | | | Y | | | N/A | | | | |  |
| Was the selection process likely to select subjects that were representative? | N | N | | | Y | | | Y | | Y | | | | Y | | | Y | | | Y | | | | | Y | | Y | | | Y | | | N | | | | |  |
| Were measures undertaken to address and categorize non-responders? | N | N | | | N | | | Y | | N | | | | N | | | N | | | Y | | | | | N | | N | | | N | | | N | | | | |  |
| Were the risk factor and outcome variables measured appropriate to the aims of the study? | Y | Y | | | Y | | | Y | | Y | | | | Y | | | Y | | | Y | | | | | Y | | Y | | | Y | | | Y | | | | |  |
| Were the risk factor and outcome variables measured correctly using instruments that had been trailed/piloted or published previously? | Y | Y | | | Y | | | N | | Y | | | | Y | | | Y | | | Y | | | | | Y | | Y | | | Y | | | N/A | | | | |  |
| Is it clear what was used to determine statistical significance and/or precision estimate? | Y | Y | | | Y | | | Y | | Y | | | | N | | | Y | | | Y | | | | | Y | | Y | | | Y | | | Y | | | | |  |
| Were methods sufficiently described to enable them to be repeated? | Y | Y | | | Y | | | Y | | Y | | | | Y | | | Y | | | Y | | | | | Y | | Y | | | Y | | | Y | | | | |  |
| Were the basic data described adequately? | Y | Y | | | Y | | | Y | | Y | | | | Y | | | Y | | | Y | | | | | Y | | Y | | | Y | | | Y | | | | |  |
| Does the response rate raise concerns about non-response-bias? | N/A | N | | | N | | | N | | N | | | | N | | | N | | | N | | | | | Y | | N | | | N | | | N | | | | |  |
| Was information on non-responders described? | N | N | | | N | | | Y | | N | | | | Y | | | N | | | Y | | | | | N | | N | | | N | | | N | | | | |  |
| Were the results internally consistent? | N/A | N/A | | | N/A | | | N/A | | Y | | | | N/A | | | N/A | | | N/A | | | | | N/A | | N/A | | | N/A | | | N/A | | | | |  |
| Were the results for analyses, described in the methods, presented? | Y | Y | | | Y | | | Y | | Y | | | | Y | | | Y | | | Y | | | | | Y | | Y | | | Y | | | Y | | | | |  |
| Were the authors’ discussions and conclusions justified by the results? | Y | Y | | | Y | | | Y | | Y | | | | Y | | | Y | | | Y | | | | | Y | | Y | | | Y | | | Y | | | | |  |
| Were the limitations of the study discussed? | N | N | | | Y | | | Y | | Y | | | | Y | | | Y | | | Y | | | | | Y | | Y | | | Y | | | Y | | | | |  |
| Were there any funding sources or COI? | Y | Y | | | N | | | Y | | N | | | | Y | | | Y | | | N | | | | | Y | | Y | | | Y | | | Y | | | | |  |
| Was ethical approval or consent of participants attained? | Y | N | | | N | | | Y | | N | | | | Y | | | N/A | | | Y | | | | | Y | | Y | | | Y | | | Y | | | | |  |
|  | Ruf et al 2008 [73] | | Schwartz et al 2003 [74] | | | Vollmar et al 2008 [75] | | | Vollmar et al 2009 [76] | | |  | | | | | | | | | | | | | | | | | | | | | | | | | | |
| Were the aims of the study clear? | Y | | Y | | | Y | | | Y | | |  | | | | | | | | | | | | | | | | | | | | | | | | | | |
| Was the design appropriate for the stated aims? | Y | | Y | | | Y | | | Y | | |  | | | | | | | | | | | | | | | | | | | | | | | | | | |
| Was the sample size justified? | N/A | | N | | | Y | | | Y | | |  | | | | | | | | | | | | | | | | | | | | | | | | | | |
| Was the target/reference population clearly defined | Y | | N | | | Y | | | Y | | |  | | | | | | | | | | | | | | | | | | | | | | | | | | |
| Was the sample taken from an appropriate population base? | Y | | N | | | Y | | | N | | |  | | | | | | | | | | | | | | | | | | | | | | | | | | |
| Was the selection process likely to select subjects that were representative? | Y | | N | | | N | | | N/A | | |  | | | | | | | | | | | | | | | | | | | | | | | | | | |
| Were measures undertaken to address and categorize non-responders? | Y | | N | | | Y | | | N | | |  | | | | | | | | | | | | | | | | | | | | | | | | | | |
| Were the risk factor and outcome variables measured appropriate to the aims of the study? | Y | | Y | | | Y | | | Y | | |  | | | | | | | | | | | | | | | | | | | | | | | | | | |
| Were the risk factor and outcome variables measured correctly using instruments that had been trailed/piloted or published previously? | Y | | N | | | Y | | | N | | |  | | | | | | | | | | | | | | | | | | | | | | | | | | |
| Is it clear what was used to determine statistical significance and/or precision estimate? | Y | | N | | | Y | | | Y | | |  | | | | | | | | | | | | | | | | | | | | | | | | | | |
| Were methods sufficiently described to enable them to be repeated? | Y | | Y | | | Y | | | Y | | |  | | | | | | | | | | | | | | | | | | | | | | | | | | |
| Were the basic data described adequately? | Y | | Y | | | Y | | | Y | | |  | | | | | | | | | | | | | | | | | | | | | | | | | | |
| Does the response rate raise concerns about non-response-bias? | N | | N | | | N | | | Y | | |  | | | | | | | | | | | | | | | | | | | | | | | | | | |
| Was information on non-responders described? | Y | | N | | | Y | | | N | | |  | | | | | | | | | | | | | | | | | | | | | | | | | | |
| Were the results internally consistent? | N/A | | N/A | | | N/A | | | Y | | |  | | | | | | | | | | | | | | | | | | | | | | | | | | |
| Were the results for analyses, described in the methods, presented? | Y | | Y | | | Y | | | Y | | |  | | | | | | | | | | | | | | | | | | | | | | | | | | |
| Were the authors’ discussions and conclusions justified by the results? | Y | | Y | | | Y | | | Y | | |  | | | | | | | | | | | | | | | | | | | | | | | | | | |
| Were the limitations of the study discussed? | Y | | Y | | | N | | | Y | | |  | | | | | | | | | | | | | | | | | | | | | | | | | | |
| Were there any funding sources or COI? | Y | | Y | | | N | | | Y | | |  | | | | | | | | | | | | | | | | | | | | | | | | | | |
| Was ethical approval or consent of participants attained? | N | | N | | | N/A | | | Y | | |  | | | | | | | | | | | | | | | | | | | | | | | | | | |

Notes: Symbols indicate: N = No, Y = Yes, N/A = not applicable, no comment
* indicates mixed methods studies, that were also appraised with the AXIS tool
